# Variable Na_v_1.5 Protein Expression from the Wild-Type Allele Correlates with the Penetrance of Cardiac Conduction Disease in the *Scn5a* ^+/−^ Mouse Model

**DOI:** 10.1371/journal.pone.0009298

**Published:** 2010-02-19

**Authors:** Anne-Laure Leoni, Bruno Gavillet, Jean-Sébastien Rougier, Céline Marionneau, Vincent Probst, Solena Le Scouarnec, Jean-Jacques Schott, Sophie Demolombe, Patrick Bruneval, Christopher L. H. Huang, William H. Colledge, Andrew A. Grace, Hervé Le Marec, Arthur A. Wilde, Peter J. Mohler, Denis Escande, Hugues Abriel, Flavien Charpentier

**Affiliations:** 1 INSERM, UMR915, l'Institut du Thorax, Nantes, France; 2 CNRS, ERL3147, Nantes, France; 3 Université de Nantes, Nantes, France; 4 CHU Nantes, l'Institut du Thorax, Nantes, France; 5 Department of Clinical Research, University of Bern, Bern, Switzerland; 6 INSERM, U652, Université Paris V, Paris, France; 7 Department of Cardiology, Academic Medical Center, Amsterdam, The Netherlands; 8 The Section of Cardiovascular Biology, Departments of Biochemistry and Physiology, University of Cambridge, Cambridge, United Kingdom; 9 Department of Internal Medicine, University of Iowa Carver College of Medicine, Iowa City, Iowa, United States of America; 10 Department of Pharmacology and Toxicology, University of Lausanne, Lausanne, Switzerland; Istituto Dermopatico dell'Immacolata, Italy

## Abstract

**Background:**

Loss-of-function mutations in *SCN5A*, the gene encoding Na_v_1.5 Na^+^ channel, are associated with inherited cardiac conduction defects and Brugada syndrome, which both exhibit variable phenotypic penetrance of conduction defects. We investigated the mechanisms of this heterogeneity in a mouse model with heterozygous targeted disruption of *Scn5a* (*Scn5a* ^+/−^ mice) and compared our results to those obtained in patients with loss-of-function mutations in *SCN5A*.

**Methodology/Principal Findings:**

Based on ECG, 10-week-old *Scn5a* ^+/−^ mice were divided into 2 subgroups, one displaying severe ventricular conduction defects (QRS interval>18 ms) and one a mild phenotype (QRS≤18 ms; QRS in wild-type littermates: 10–18 ms). Phenotypic difference persisted with aging. At 10 weeks, the Na^+^ channel blocker ajmaline prolonged QRS interval similarly in both groups of *Scn5a* ^+/−^ mice. In contrast, in old mice (>53 weeks), ajmaline effect was larger in the severely affected subgroup. These data matched the clinical observations on patients with *SCN5A* loss-of-function mutations with either severe or mild conduction defects. Ventricular tachycardia developed in 5/10 old severely affected *Scn5a* ^+/−^ mice but not in mildly affected ones. Correspondingly, symptomatic *SCN5A*–mutated Brugada patients had more severe conduction defects than asymptomatic patients. Old severely affected *Scn5a* ^+/−^ mice but not mildly affected ones showed extensive cardiac fibrosis. Mildly affected *Scn5a* ^+/−^ mice had similar Na_v_1.5 mRNA but higher Na_v_1.5 protein expression, and moderately larger I_Na_ current than severely affected *Scn5a* ^+/−^ mice. As a consequence, action potential upstroke velocity was more decreased in severely affected *Scn5a* ^+/−^ mice than in mildly affected ones.

**Conclusions:**

*Scn5a* ^+/−^ mice show similar phenotypic heterogeneity as *SCN5A*-mutated patients. In *Scn5a* ^+/−^ mice, phenotype severity correlates with wild-type Na_v_1.5 protein expression.

## Introduction

The cardiac voltage-gated Na^+^ channel Na_v_1.5, encoded by *SCN5A* gene, determines the upstroke velocity of the action potential and, in conjunction with gap junction channels and the organization of the collagenous skeleton, controls the propagation of the cardiac electrical impulse. Loss-of-function mutations of *SCN5A* are associated to the inherited form of progressive cardiac conduction defect [1], [2] (PCCD) and to the Brugada syndrome (BrS) [3]–[5]. In patients with inherited PCCD, the conduction of the cardiac impulse is abnormally slow and becomes progressively slower with aging leading ultimately to atrioventricular block and pacemaker implantation in the elderly [2]. The presence of conduction defects is also one striking feature of BrS *SCN5A*-mutation carriers [6], [7]. As for most inherited electrical cardiac diseases, penetrance of the defect caused by *SCN5A* haploinsufficiency is variable among carriers of the same mutation, with patients showing severe conduction alterations and others exhibiting an almost normal phenotype [2]. This demonstrates that factors independent of the morbid gene exert considerable influence on outcome of the inherited “channelopathy”. One obvious possibility in humans is the contribution of “modifier genes”, which could control expressivity of the monogenic defect [8].

A mouse model with targeted disruption of *Scn5a* has been established [9]. In previous works [10], [11], we showed that heterozygous *Scn5a* deficient (*Scn5a* ^+/−^) mice convincingly recapitulate PCCD phenotype including progressive impairment of atrial and ventricular conduction with aging. Because progressive decline in conduction velocity is also associated with myocardial rearrangements and fibrosis, we provided the first demonstration that an isolated ion channel defect can lead to myocardial structural anomalies [10]. In the present study, we demonstrate that: (i) the expression of the conduction anomalies is variable among inbred *Scn5a* ^+/−^ mice; (ii) *Scn5a* ^+/−^ mice with severe conduction defects (severe phenotype) exhibit more myocardial rearrangements with aging than mice with mild conduction defects (mild phenotype); (iii) old mice with a severe, but not with a mild phenotype, have a markedly reduced conduction reserve and show spontaneous ventricular arrhythmias; (iv) similarly, symptomatic BrS patients carrying *SCN5A* mutations have more pronounced conduction slowing than asymptomatic patients; and, (v) in mice, the expression of the phenotype correlates with the ability of the normal allele to produce at the post-transcriptional level variable amount of functional Na_v_1.5 channel proteins.

## Results

### Heterogeneity of the Intraventricular Conduction Defect in S*cn5a* ^+/−^ Mice

**Figure 1:**
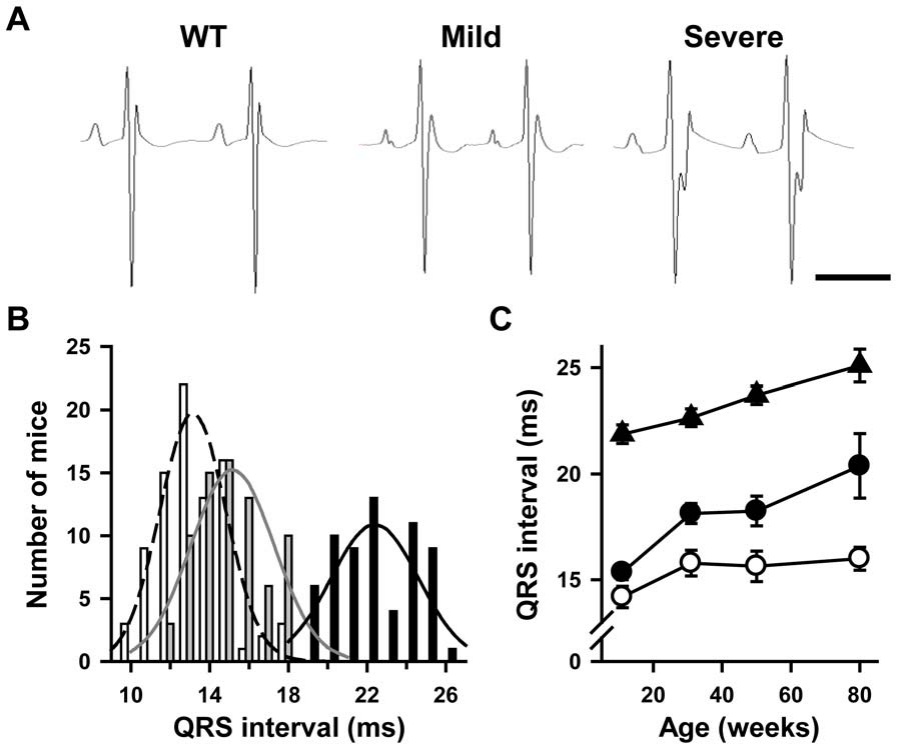
Variable degrees of ventricular conduction defects in *Scn5a* ^+/−^ mice. **A.** Representative lead I ECGs from 10 week-old wild-type (WT) mice and *Scn5a* ^+/−^ mice with mild and severe conduction defects. Scale bar, 100 ms. **B.** Distribution of QRS interval duration with corresponding Gaussian fits in 10 week-old WT mice (white bars) and *Scn5a* ^+/−^ mice with mild (grey bars) and severe (black bars) phenotype. **C.** Effects of age (X-axis) on QRS interval duration (Y-axis) in WT mice (open symbols) and *Scn5a* ^+/−^ mice with mild (filled circles) and severe (filled triangles) phenotype. See Table 1 for statistics.

Young adult (10–12 week-old) *Scn5a* ^+/−^ mice were investigated for their ventricular conduction parameters. Figure 1A shows representative ECG recordings from a WT and two *Scn5a* ^+/−^ mice. The two *Scn5a* ^+/−^ mice clearly differed since one exhibited a markedly prolonged QRS complex whereas the other had a relatively normal one. Figure 1B displays the QRS interval distribution in 84 WT littermates and 136 *Scn5a* ^+/−^ young adult mice. Statistical analysis showed that the QRS interval duration was not normally distributed in the *Scn5a* ^+/−^ population but distributed according to two subgroups, (i) one with a severe conduction phenotype (QRS>18 ms; mean QRS = 22±0 ms; range 19–26 ms; n = 63), and (ii) the other with a mild conduction phenotype (QRS≤18 ms; mean QRS = 15±0 ms; range 12–18 ms; n = 73; p<0.05 *versus* WT and severe phenotype). In the WT littermate population, QRS duration ranged from 10 to 18 ms (mean QRS = 13±0 ms). Each group (WT, mild and severe) differed significantly from the two others with p values <0.001. Phenotype severity in *Scn5a* ^+/−^ mice was not influenced by gender (male/female ratio: 43/30 and 31/32 in mice with mild and severe conduction defects, respectively; NS). Moreover, among the 25 litters in which all *Scn5a* ^+/−^ mice have been phenotyped, the average proportion of mice with mild conduction defects was 0.53±0.05 (NS *versus* theoretical proportion of 0.5). In 2 litters, all *Scn5a* ^+/−^ mice had mild conduction defects (2 and 3 mice, respectively). In 2 others, all *Scn5a* ^+/−^ mice had, in contrast, severe conduction defects (3 mice in each). Thus, based on the QRS duration, we distinguished 3 groups of mice: WT mice, *Scn5a* ^+/−^ mice with a mild phenotype, *i.e.* with a small QRS prolongation, and *Scn5a* ^+/−^ mice with a severe phenotype, *i.e.* with a marked QRS prolongation.

Subpopulations of these 3 groups were included in a longitudinal study conducted up to the age of 80 weeks. There was a progressive increase in QRS interval with age in WT and *Scn5a* ^+/−^ mice, although this increase was slightly more pronounced in *Scn5a* ^+/−^ mice, thus confirming previous results (Figure 1C) [10]. Among *Scn5a* ^+/−^ mice, the difference in phenotype was maintained over time: each mouse remained in its initial group. Atrial and atrioventricular conduction (as evaluated respectively by P wave and PR interval durations) were similar in the two groups of *Scn5a* ^+/−^ mice and significantly slower than in WT mice (Table 1).

**Table 1:** ECG characteristics of wild-type (WT) and *Scn5a* ^+/−^ mice with mild and severe phenotype.

	n	Age (weeks)	RR (ms)	P (ms)	PR (ms)	QRS (ms)	QT (ms)	QTc
**WT**	16	11±0	128±3	15±0	35±1	14±1	64±1	58±1
	14	31±0	122±3	16±1	34±1	16±1	60±2	54±1
	14	50±1	122±3	16±1	35±1	16±1	58±1	53±1
	12	81±3	115±1^§§§^	15±1	34±1	16±1	58±1^§^	54±1
**Mild ** ***Scn5a*** **^+/−^**	20	12±1	135±3	18±1**	39±1***	15±0	67±1	58±1
	16	31±0	127±2	18±1	39±1***	18±0**^§§^	61±2^§§^	54±1^§§^
	16	49±1	130±3	18±0*	40±1***	18±1**^§§§^	61±1^§§^	53±1^§§^
	8	78±5	132±2**	19±1*	42±2***	20±2***^§§§^	66±2**	57±1
**Severe ** ***Scn5a*** **^+/−^**	17	11±1	142±4***	18±0*	39±1***	22±0***^†^	69±1*	59±1
	17	32±0	128±2^§§^	19±1*	38±1***	23±0***^†^	63±1^§§^	55±1
	17	50±2	124±2^§§§^	18±0***	39±1***	24±0***^†^	62±1^§§^	56±1
	10	80±3	128±2*	19±1**	43±1***^§§^	25±1***^†§§^	68±2***	59±1*

Abbreviations: n, number of animals per group; RR, RR interval duration; P, P wave duration; PR, PR interval duration; QRS, QRS complex duration; QT, QT interval duration; QTc, corrected QT interval duration.

Data are mean ± sem. *, **, ***, p<0.05, p<0.01 and p<0.001 respectively *versus* WT (matching age); ^†^, p<0.001 *versus* Mild (matching age); ^§^, ^§§^, ^§§§^, p<0.05, p<0.01 and p<0.001 respectively *versus* youngest age in each group.

### Reduced Conduction Reserve in S*cn5a* ^+/−^ Mice and Patients with *SCN5A* Mutations with a Severe Phenotype

**Figure 2:**
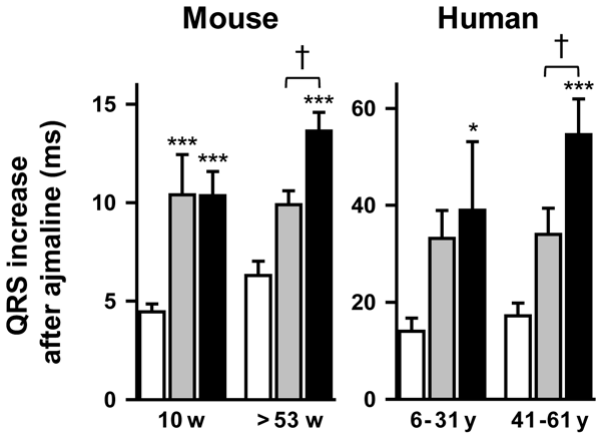
Variable effects of Na^+^ channel blockade in *Scn5a* ^+/−^ mice and *SCN5A*-mutated patients. Ajmaline-induced increase in QRS interval (Y-axes) in 10 week- and >53 week-old WT (n = 11 and n = 10; open bars), mildly (n = 10 and n = 10; grey bars) and severely (n = 11 and n = 10; black bars) affected *Scn5a* ^+/−^ mice (left panel), and in young (6–31 years; mean = 22 years) and older (41–61 years; mean = 50 years) non-mutated (n = 18 and n = 17, open bars) and *SCN5A*-mutated patients with either mild (n = 12 and n = 11; grey filled bars) or severe (n = 7 and n = 12; black filled bars) QRS interval prolongation (right panel). *, ***, p<0.05 and p<0.001 respectively *versus* WT mice or non-mutated patients. †, p<0.05 *versus Scn5a* ^+/−^ mice or *SCN5A*-mutated patients with a mild phenotype. The difference between old mildly affected mice and old WT mice did not reach significance (p = 0.07). Same comment for the older patients.

To estimate their conduction reserve, we challenged young (10 weeks) and old (>53 weeks) *Scn5a* ^+/−^ mice with the Na^+^ channel blocker ajmaline (20 mg/kg i.p.). As shown in Figure 2, in young mice, the effects of ajmaline on the QRS interval were modest and similar in the two subgroups of *Scn5a* ^+/−^ mice (+10.4±2.0 ms *versus* +10.4±1.2 ms in mice with mild and severe phenotype, respectively). In old mice, ajmaline prolonged more the QRS interval in animals with a severe phenotype (+12.0±0.8 ms) than in animals with a mild phenotype (+9.6±0.7 ms; p<0.05).

These mouse data were similar to the results of ajmaline tests performed in patients from Nantes cohort diagnosed with either PCCD or BrS and carrying mutations on *SCN5A* gene. These patients were separated in two groups based on the duration of QRS interval under baseline conditions: one with mild conduction defects (baseline QRS≤108 ms) and one with severe conduction defects (QRS>108 ms; see methods section for the cutoff value definition). As shown in Figure 2B, the ajmaline-induced QRS prolongation in aged patients was more pronounced in those with severe conduction defects under baseline conditions (+55±7 ms) than in those with mild conduction defects (+34±5 ms; p<0.05). In contrast, no statistical difference was seen in the younger patients.

Thus, the conduction reserve is reduced with aging both in mice and humans with markedly prolonged QRS interval.

### Extensive Cardiac Fibrosis in the Ventricular Myocardium of *Scn5a* ^+/−^ Mice with a Severe Phenotype

**Figure 3:**
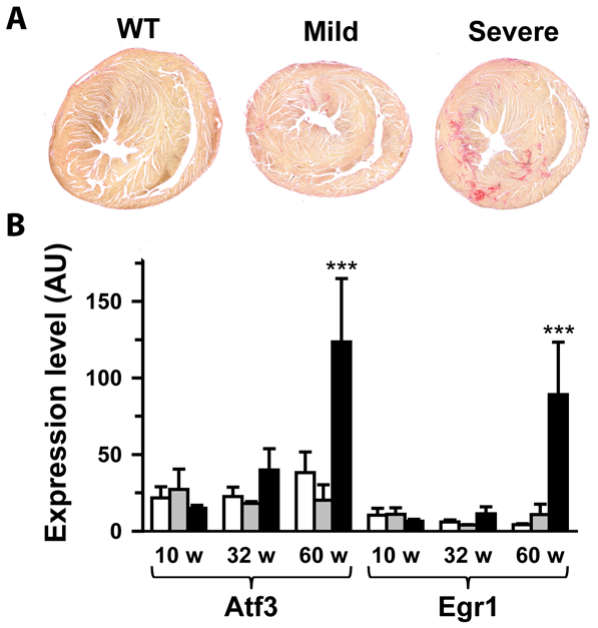
Variable levels of fibrosis in *Scn5a* ^+/−^ mice. **A.** Sirius red staining of ventricle from 85 week-old WT, mildly and severely affected *Scn5a* ^+/−^ mice. Fibrosis appears in red. A score of 0 was attributed to the WT mouse shown, 1 to the mild *Scn5a* ^+/−^ mouse and respectively 2 and 3 to left and right severe *Scn5a* ^+/−^ mice. **B.** *Atf3* and *Egr1* mRNA ventricular levels (in arbitrary units) in WT (open bars), mildly (grey bars) and severely (black bars) affected *Scn5a* ^+/−^ mice as a function of age. ***, p<0.001 *versus* WT and mild.

Previously, we showed that heterozygous *Scn5a* invalidation in mouse produces ventricular rearrangements and fibrosis with aging [10], [11]. We also showed that two transcription factors, *Atf3*, a stress-inducible gene, and *Egr1*, *an* early growth response gene, were up-regulated with aging in *Scn5a* ^+/−^ mice. Ventricular sections were evaluated for the presence of fibrosis in 83±3 week-old mice. The investigator was blinded to genotype and phenotype. *Scn5a* ^+/−^ mice with a severe phenotype were characterized by extensive fibrosis located in the left ventricular free wall and intraventricular septum (Figure 3A). In contrast, fibrosis in *Scn5a* ^+/−^ mice with a mild phenotype when present was restricted to small areas. Semi-quantitative assessment of cardiac fibrosis led to mean scores of 1.88±0.40 in *Scn5a* ^+/−^ mice with severe phenotype (n = 8; p<0.05 *versus* WT), 0.60±0.40 in *Scn5a* ^+/−^ mice with mild phenotype (n = 5) and 0.25±0.25 in WT mice (n = 4). No fibrosis was observed in the right ventricular outflow tract region of *Scn5a* ^+/−^ mice with a severe phenotype (not shown). The transcription factors *Atf3* and *Egr1* were exclusively over-expressed in old *Scn5a* ^+/−^ mice with a severe phenotype (Figure 3B).

### Ventricular Arrhythmias in S*cn5a* ^+/−^ Mice with a Severe Phenotype and Wider QRS Interval in Symptomatic *SCN5A*-Related BrS Patients

**Figure 4:**
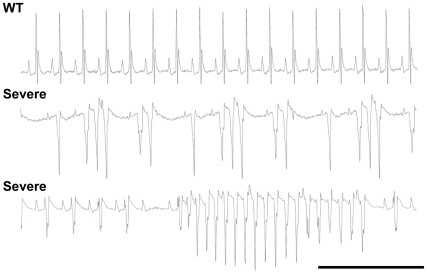
Cardiac arrhythmias in *Scn5a* ^+/−^ mice with severe phenotype. Representative episodes of spontaneous ventricular arrhythmias recorded in two different *Scn5a* ^+/−^ mice with a severe phenotype under anesthesia. An ECG recorded in an age-matched WT mouse is given for comparison. Scale bar, 500 ms.

With aging, *Scn5a* ^+/−^ mice with a severe phenotype had an increased propensity to develop spontaneous ventricular arrhythmias under anesthesia (5/10) in comparison to *Scn5a* ^+/−^ mice with a mild phenotype (0/8; p<0.05) and WT mice (0/12; p<0.05). The severity of the arrhythmias ranged from ventricular extrasystoles to salvoes of ventricular tachycardia (Figure 4).

Patients from Nantes database with *SCN5A*-related BrS were pooled with their counterparts from Amsterdam database. Symptomatic patients, defined as having a clinical history of collapses, syncopes, documented ventricular tachycardia (and/or fibrillation) or aborted sudden death, were characterized by a longer QRS interval than asymptomatic patients (Table 2).

**Table 2:** ECG characteristics of asymptomatic (n = 84) and symptomatic (n = 24) *SCN5A*-mutated Brugada patients.

	Age (yrs)	RR (ms)	P (ms)	PR (ms)	QRS (ms)	QT (ms)	QTc
**Asymptomatic**	41±2	901±18	93±3	191±4	110±2	393±3	417±3
**Symptomatic**	46±3	915±31	97±4	200±5*	118±4*	401±6	417±7

Same abbreviations as in Table 1.

Data are mean ± sem. *, p<0.05 *versus* asymptomatic patients.

### Differential Transcriptional Remodeling between Mild and Severe Phenotype *Scn5a* ^+/−^ Mice

**Figure 5:**
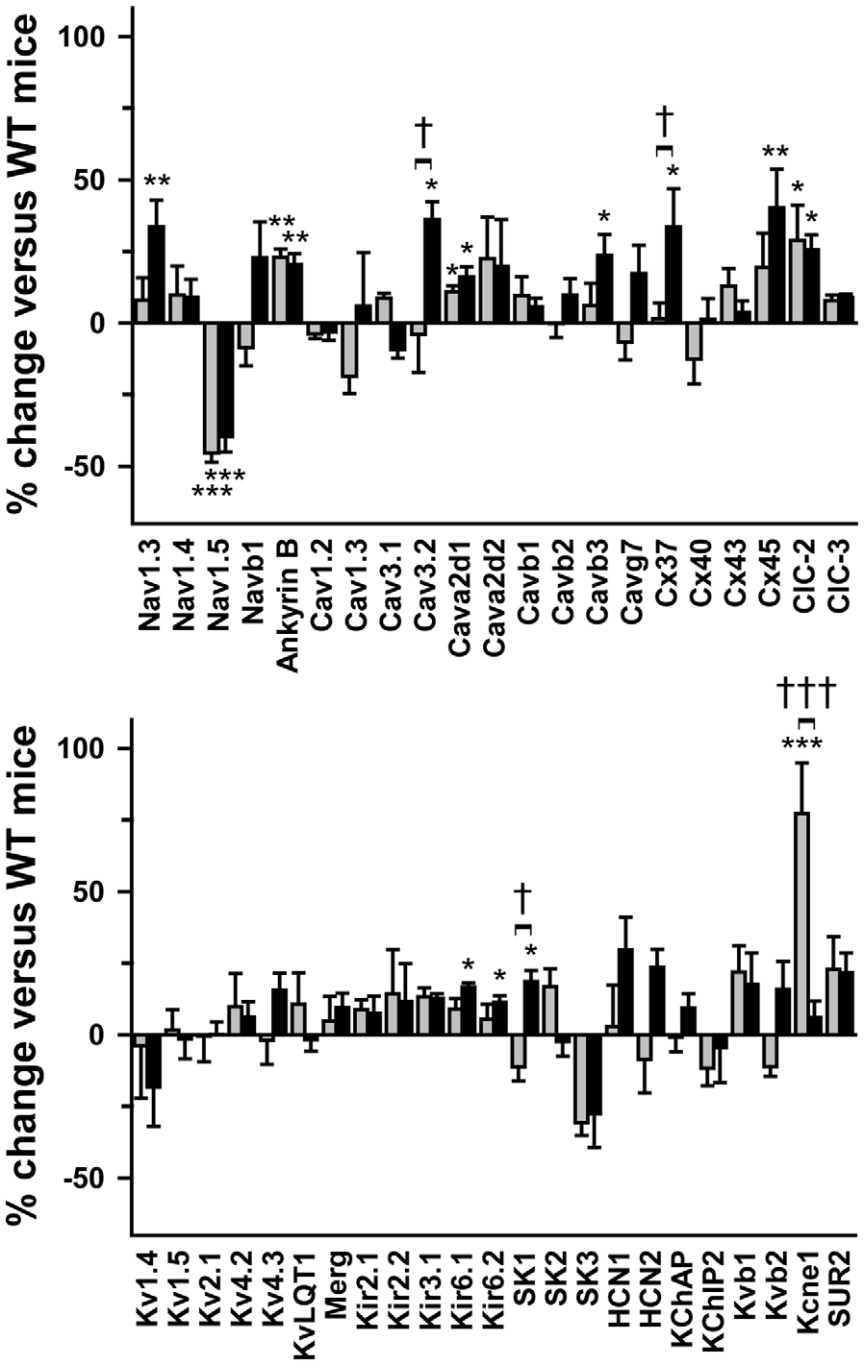
Moderate ionic remodeling in *Scn5a* ^+/−^ mice. Percentage of variation in ventricular expression (Y-axes) of 46 genes encoding ion channel subunits (ch) and connexins (Cx) in *Scn5a* ^+/−^ mice with mild (grey bars; n = 5) and severe (black bars; n = 7) phenotype *versus* WT mice (n = 10). Sub, subunits. *, **, ***, p<0.05, p<0.01 and p<0.001 respectively *versus* WT; †, †††, p<0.05 and p<0.001 respectively *versus* mild phenotype.

We then investigated the molecular mechanisms potentially accounting for variable penetrance in *Scn5a* ^+/−^ mice. We first hypothesized that molecular remodeling of ion channel gene expression could variably modulate the deficit. Figure 5 shows variations of expression of 46 ion-channel subunits in the ventricles of 10 week-old *Scn5a* ^+/−^ mice relative to their expression in WT littermates. Na_v_1.5 transcripts were similarly down-regulated by almost 50% in *Scn5a* ^+/−^ mice with both mild and severe phenotype, showing that the phenotype heterogeneity was not due to differential transcription of the WT *Scn5a* allele. Ion channel remodeling in *Scn5a* ^+/−^ mice was limited to 10 genes. Among them, 4 were moderately up-regulated in both groups of *Scn5a* ^+/−^ mice including ankyrin-B, an anchoring protein involved in ion channel (including Na_v_1.5) and transporter targeting [12], [13]. Few genes were differentially regulated among the *Scn5a* ^+/−^ mice. Five were up-regulated only in severely affected *Scn5a* ^+/−^ mice, including genes encoding the Na^+^ channel α-subunit Na_v_1.3, the T-type Ca^2+^ channel α-subunit Ca_v_3.2 and connexin 37. Finally, *Kcne1*, which encodes KvLQT1 β-subunit, was up-regulated only in mice with mild phenotype. No gene expression (other than *Scn5a*) was significantly down-regulated. The expression of genes encoding pumps and exchangers was not altered.

### Reduced Na_v_1.5 Protein Expression and I_Na_ Density in *Scn5a* ^+/−^ Mice with a Severe Phenotype

We then investigated Na_v_1.5 protein expression. Investigators were blinded to the genotype and phenotype for all the experiments presented below.

**Figure 6:**
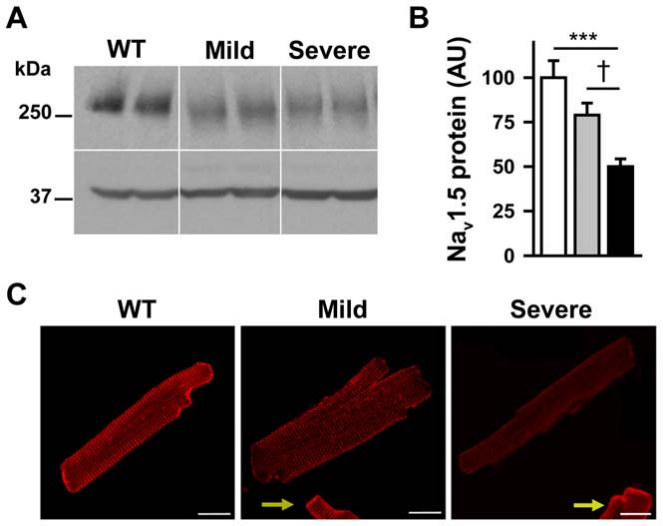
Decreased Na_v_1.5 expression in *Scn5a* ^+/−^ mice. **A.** Representative Western blots showing the expression levels of Na_v_1.5 in WT mice and *Scn5a* ^+/−^ mice (mean age = 18±1 weeks) with a mild or a severe phenotype. Protein loading was controlled by anti-actin immunoblotting. **B.** Quantification of Na_v_1.5 expression in WT mice (open bars) and *Scn5a* ^+/−^ mice with mild (grey bars) and severe (black bars) phenotype was performed on Western blots from 10 mice in each group by normalizing the intensities of the Na_v_1.5 bands to the actin bands. ***, p<0.001 *versus* WT; †, p<0.05 *versus* mild *Scn5a* ^+/−^ mice. **C.** Confocal images of Na_v_1.5 in ventricular cardiomyocytes isolated from WT mice (left panel) and *Scn5a* ^+/−^ mice with mild (middle panel) and severe (right panel) phenotype. Scale bar, 20 µm. In *Scn5a* ^+/−^ images presented, a WT cardiomyocyte is also included in the image frame (yellow arrows) to illustrate the clear difference in WT *versus Scn5a* ^+/−^ intercalated disc staining intensities.

In young *Scn5a* ^+/−^ mice, global Na_v_1.5 protein was reduced by 50±4% in ventricular lysates of animals with a severe phenotype *versus* WT littermates (p<0.001) and by only 21±7% for animals with a mild phenotype (p<0.05 *versus* severe phenotype, NS *versus* WT; Figure 6).

At the single cell level, immunofluorescence and confocal imaging experiments demonstrated that Na_v_1.5 staining intensity in intercalated discs was reduced by 42±4% in myocytes from mice with severe phenotype (66 cells from 3 mice; p<0.01 *versus* WT), but only by 30±2% for mice with mild phenotype (60 cells from 3 mice; p<0.05 *versus* severe, p<0.01 *versus* WT; Figure 6C).

**Figure 7:**
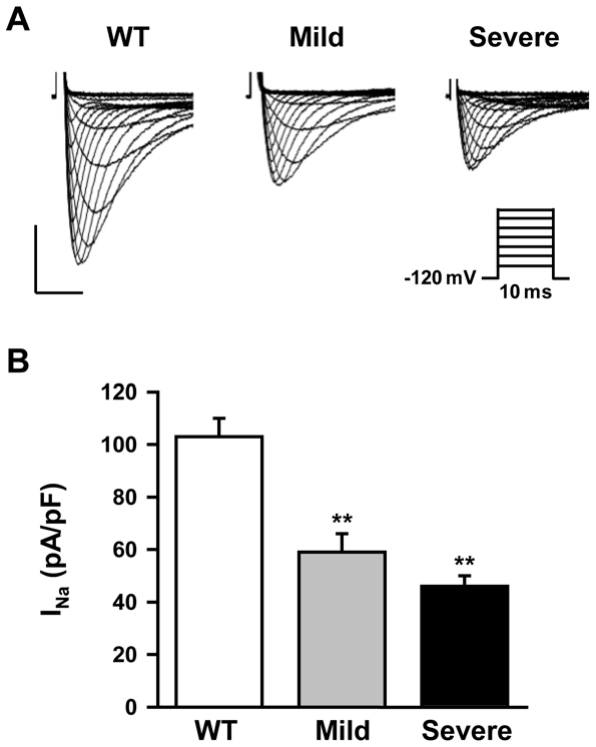
Variable I_Na_ densities in *Scn5a* ^+/−^ single ventricular cardiomyocytes. **A.** Representative I_Na_ traces (protocol in *inset*) obtained from ventricular myocytes in a 12 week-old WT mouse and *Scn5a* ^+/−^ mice with mild and severe phenotype. Horizontal bar, 2 ms; vertical bar, 40 pA/pF. **B.** I_Na_ density in myocytes from WT and *Scn5a* ^+/−^ mice with mild and severe phenotype (4 mice in each group, 4 to 6 cells for each mouse). **, p<0.01 *versus* WT mice.

Accordingly, the I_Na_ density was 28% larger in mice with a mild phenotype (59±7 pA/pF; 18 cells from 4 mice) than in mice with a severe phenotype (46±4 pA/pF; 21 cells from 4 mice; Figure 7) although this difference did not reach significance (p = 0.08). For comparison, amplitude of I_Na_ in WT mice was 103±7 pA/pF (16 cells from 4 mice). In summary, the three independent techniques indicated a less severe reduction of Na_v_1.5 proteins in mice with a mild phenotype than in mice with a severe phenotype. We also observed a shift towards more positive voltage values of steady-state inactivation and activation curves in *Scn5a* ^+/−^ mice *versus* WT mice (Table 3). However, no difference was observed among the *Scn5a* ^+/−^ mice.

**Table 3:** I_Na_ steady-state activation and inactivation properties of isolated ventricular myocytes from wild-type (WT) and *Scn5a* ^+/−^ mice with mild and severe phenotype.

	WT	Mild *Scn5a* ^+/−^	Severe *Scn5a* ^+/−^
**Activation**	n = 12	n = 13	n = 10
**K (mV/e-fold)**	5.9±0.1	6.1±0.2	5.9±0.1
**V_1/2_ (mV)**	−37.6±1.0	−31.2±2.0*	−31.0±1.3*
**Inactivation**	n = 10	n = 10	n = 10
**K (mV/e-fold)**	6.4±0.2	6.8±0.3	7.0±0.2
**V_1/2_ (mV)**	−77.9±2.5	−68.8±2.5*	−68.0±1.6**

Abbreviations are: n, number of myocytes per group; K, slope factor; V_1/2_, half-activation or half-inactivation voltage (see methods section for calculation procedure).

Data are mean ± sem. *, **, p<0.05 and p<0.01 respectively versus WT (Kruskal-Wallis test, Dunn's post-test).

**Figure 8:**
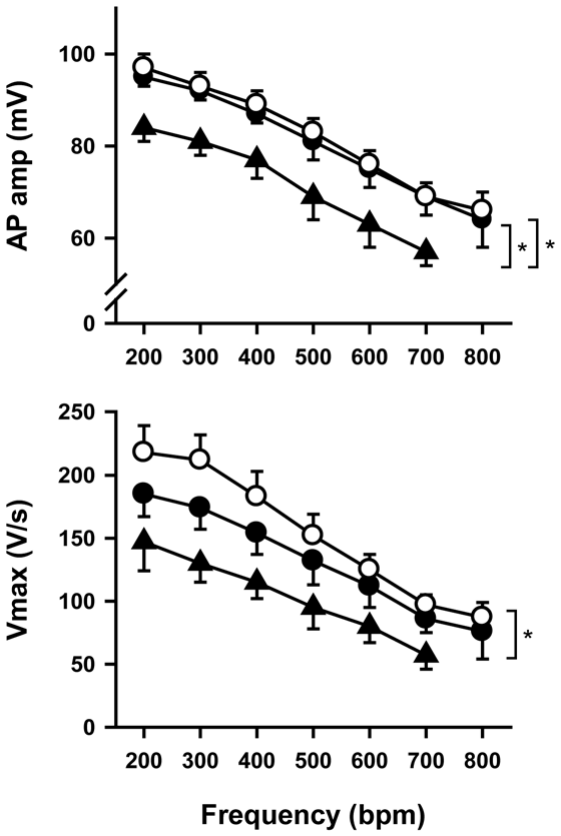
Action potential amplitude (AP amp; top) and maximum upstroke velocity (Vmax; bottom) as a function of pacing frequency. White circles, wild type mice (n = 7); black circles, *Scn5a* ^+/−^ mice with a mild phenotype (n = 6); black triangles, *Scn5a* ^+/−^ mice with a severe phenotype (n = 5). At 800 bpm, 4 preparations from *Scn5a* ^+/−^ mice with a severe phenotype were periodically (for 1 of them) or continuously (for the 3 others) in 2∶1 conduction block. *, p<0.05.

The decrease in I_Na_ induced a moderate (11–15%) non-significant decrease in maximum upstroke velocity (V_max_) of ventricular action potentials in *Scn5a* ^+/−^ mice with mild conduction defects (Figure 8). In contrast, V_max_ was significantly decreased by 33–42% in *Scn5a* ^+/−^ mice with a severe phenotype. They also exhibited a significant decrease in action potential amplitude. Other action potential parameters did not differ among the groups.

## Discussion

The main findings of the present study are: (i) *Scn5a* ^+/−^ mice exhibit variable penetrance of the conduction deficit, as do patients with *SCN5A* loss-of-function mutations [2], [6]; (ii) *Scn5a* ^+/−^ mice with severe ventricular conduction defects, exhibit more myocardial rearrangements with aging than mice with a mild phenotype; (iii) only old mice with a severe phenotype have a markedly reduced conduction reserve and show spontaneous ventricular arrhythmias; (iv) symptomatic BrS patients carrying *SCN5A* mutations have more pronounced conduction slowing than asymptomatic patients; and (v) the expressivity of the conduction deficit in *Scn5a* ^+/−^ mice is correlated with the expression level of WT functional Na_v_1.5 proteins. Mice with a severe phenotype correspond to the expected haploinsufficient situation since they exhibit ≈50% reduction in Na_v_1.5 protein expression whereas Na_v_1.5 reduction in mice with a mild phenotype was less pronounced.

Because distribution of QRS intervals in the *Scn5a* ^+/−^ mouse population is bimodal, we speculate that a compensatory mechanism is active in the mild-phenotype subgroup but inactive in the severe-phenotype subgroup. Such a mechanism governs the severity of the conduction slowing that in turn commands the extent of myocardial rearrangements and fibrosis, confirming our hypothesis of a direct link between ventricular conduction defects and fibrosis [10]. Moreover, that occurrence of large areas of fibrosis in mice with a severe phenotype was preceded by large overexpression of *Egr1* and *Atf3* in these mice and not in mice with a mild phenotype, which have much less fibrosis, confirms our hypothesis that these transcription factors are involved in myocardial rearrangements [10].

### Molecular Mechanism for Variable Penetrance of the Phenotype in the Mouse

Because cardiac conduction relies not only on Na^+^ current, but also on cellular coupling and myocardial architecture, different hypotheses could be evoked to explain *Scn5a* ^+/−^ mice phenotypic variability. Variable myocardial rearrangement was not expected to explain phenotypic variability since heterogeneity is already present in young adult mice whereas fibrosis occurs only in older mice [10], [11]. We first hypothesized that Na_v_1.5 invalidation could lead to altered expression of other ion channel subunits and of connexins that would vary among mice. Our data indicate that ion channel remodeling was limited to only a few genes. Among them, some were indeed differentially regulated depending on the phenotype severity. However, the contribution of these genes to the phenotype is unlikely since they are either not or weakly expressed in adult mouse ventricle. For instance, the cardiac expression of connexin-37 is restricted to endothelial cells [14]. Overexpression of the mRNA of neuronal Na^+^ channel, Na_v_1.3, and T-type Ca^2+^ channel, Ca_v_3.2, may result from a feedback mechanism compensating for the larger conduction deficit of mice with a severe phenotype. In any case, the putative resulting increase in Na^+^ and T-type Ca^2+^ currents would improve conduction and not further deteriorate it. The consequences of KvLQT1 β-subunit (*Kcne1* gene) overexpression in *Scn5a* ^+/−^ mice with a mild phenotype are also difficult to predict. In adult mice, *Kcne1* cardiac expression is restricted to the conduction system [15]. Based on studies performed on Langendorff-perfused hearts showing that targeted disruption of *Kcne1* can alter ventricular conduction velocity [16], one could speculate that *Kcne1* overexpression in *Scn5a* ^+/−^ mice with a mild phenotype could contribute to their shorter QRS interval. However ECG recordings in *Kcne1* knockout mice did not display any alteration of the QRS interval [15], suggesting that this protein is not playing a major role in cardiac impulse propagation.

An alternative hypothesis for explaining phenotypic variability was that *Scn5a* ^+/−^ mice had variable reduction in Na^+^ current. *Scn5a* transcript levels were similar in both groups of *Scn5a* ^+/−^ mice suggesting the same transcriptional regulation of the WT. However, Na_v_1.5 protein amounts and I_Na_ were larger in *Scn5a* ^+/−^ mice with a mild phenotype. The reason for increased expression of Na_v_1.5 protein in mice with a mild phenotype is as yet unknown. In addition to gene transcription and RNA processing, the expression of functional Na_v_1.5 proteins will depend on a series of complex interacting processes such as protein synthesis, assembly and post-translational modifications, trafficking to the sarcolemma, anchoring to the cytoskeleton and regulation of endocytosis and degradation, which are yet incompletely understood [17], [18].

At first glance, QRS variability might appear unexpectedly large for an inbred mouse strain. It is widely recognized that environmental, investigator-dependent, and time variables can be confounding factors influencing the characterization of mouse phenotypes [19], [20]. However, some of these variables are unlikely to explain phenotypic variability of *Scn5a* ^+/−^ mice. Indeed, a single investigator carried out all ECG recordings and analyses. Moreover, since long-term follow-up studies have shown that the mice keep their phenotype severity throughout their life, possible interference with the season or time of the day can be excluded. Finally, and perhaps more importantly, histological studies, immunoblot experiments, patch-clamp and microelectrode recordings were performed under blind conditions.

In both groups of *Scn5a* ^+/−^ mice, the steady-state inactivation and activation and curves were shifted towards more positive voltages than in wild-type mice. To our knowledge, this phenomenon has not described before. It may be speculated that a decrease in the number of sarcolemmal Na^+^ channels could trigger changes in the expression/interaction of proteins that remain to be indentified altering Na_v_1.5 biophysical properties. Whatever its mechanism, one consequence of this shift should be that the fraction of Na^+^ channels available for cardiac depolarization is larger in *Scn5a* ^+/−^ mice (about 80%) than in wild-type mice (50%).

### Potential Implications in PCCD and Brugada Syndrome

We are not aware of long-term individual follow-up data in patients with PCCD that would extend over decades. Comparison between mice and humans is thus difficult to achieve. Our study points to a key similarity between *Scn5a* ^+/−^ mice and loss-of-function *SCN5A* mutation human carriers. Young *Scn5a* ^+/−^ mice or patients with markedly prolonged QRS interval have a conduction reserve similar to *Scn5a* ^+/−^ mice or patients with mildly prolonged QRS interval, but this reserve fades out with aging. If findings obtained in mouse can be transposed in human, they suggest that depending on the duration of the QRS complex at young age, one may predict the evolution of the conduction disease and requirement for pacemaker in patients with inherited PCCD. Interestingly, in families with *SCN5A*-related PCCD, the phenotype heterogeneity is high at middle age and some old mutated patients do not develop the disease [2]. In *Scn5a* ^+/−^ mouse, decreased conduction reserve is due to fibrosis and connexin expression remodeling [10], [11], which can be considered as possible therapeutic targets to limit the evolution of the disease. However, even if mice and humans present similar phenotypes, this does not imply that the same pathophysiological mechanisms are involved.

In old *Scn5a* ^+/−^ mice with severe phenotype, fibrosis and connexin expression remodeling could partly explain the high incidence of spontaneous ventricular arrhythmias, which were never observed in mice with a mild phenotype and WT mice. Indeed, the presence of large areas of fibrosis associated with connexin-43 redistribution in areas surrounding fibrosis [11] most likely contributes to the occurrence of reentrant arrhythmias. We never observed any arrhythmias in younger animals, although *ex vivo* experiments had shown that young adult *Scn5a* ^+/−^ mice were more prone than WT mice to develop arrhythmias under programmed electrical stimulation [9]. This suggests that the decrease in I_Na_ *per se* is not enough to trigger spontaneous arrhythmias in mice.

In our cohort, symptomatic *SCN5A*-related BrS patients were characterized by more pronounced ventricular conduction defects than asymptomatic patients. This confirms a recent publication by Junttila and co-workers [21] and supports further the importance of conduction defects in Brugada syndrome pathophysiology [22], [23]. Whether the substrate for arrhythmias in *SCN5A*-related BrS patients relies essentially on Na_v_1.5 dysfunction or involves additional myocardial rearrangements is unclear. Although structural abnormalities are not detected using routine noninvasive diagnostic tools, fatty replacement, right ventricular fibrosis, myocyte degeneration and apoptosis have been reported [23]–[25]. Moreover, a boy with compound heterozygosity for two *SCN5A* mutations exhibited severe degenerative changes in the ventricular conduction system [26]. Taken together, studies in patients and in *Scn5a* ^+/−^ mice strongly suggest that a primary abnormality in Na_v_1.5 channel may lead to cellular damage. On this basis, arrhythmias may occur when a sufficient degree of myocardial rearrangement has been reached. This would explain why, in the context of an inborn defect, several years (in humans) or months (in mice) might elapse before the first arrhythmic event.

## Materials and Methods

### Ethics Statement

All the animal experiments were performed in the animal facility (*Unité de Thérapeutique Expérimentale*) *and* the Inserm UMR915 laboratory which have been accredited by the French Ministry of Agriculture. Experimental procedures were approved by the regional ethic committee (*CREEA – Pays de la Loire*).

Studies involving human participants were conducted according to the French and Dutch guidelines for genetic research. All tests that were performed were approved by the medical ethical review committees of the two hospitals involved: Academic Medical Center, Amsterdam, The Netherlands; *Centre Hospitalo-Universitaire*, Nantes, France (*CCPPRB des Pays de la Loire*). Written informed consent was obtained from the patients.

### Electrocardiography in Mouse

All mice with 129/Sv genetic background were bred at *l'institut du thorax* (Nantes, France) and genotyped by polymerase chain reaction (PCR) as previously described [9]. All experiments were performed on age-matched wild-type (WT) and heterozygous littermates.

Six-lead ECGs were recorded on mice anesthetized with etomidate (25 mg/kg i.p.) and analyzed as previously described [10]. Pharmacological challenge with the Na^+^ channel blocker ajmaline (20 mg/kg, i.p.) was performed in 10 and >53 week-old mice. QRS interval lengthening was assessed 10 minutes after injection.

### ECG Recordings and Clinical Data from Patients with *SCN5A* Mutations

The study was conducted according to the French and Dutch guidelines for genetic research. All tests that were performed were approved by the medical ethical review committees of the two hospitals involved: Academic Medical Center, Amsterdam, The Netherlands; *Centre Hospitalo-Universitaire*, Nantes, France (*CCPPRB des Pays de la Loire*). Written informed consent was obtained from the patients.

A first study was aimed at evaluating the effects of ajmalin on conduction defects in patients from the Nantes database carrying *SCN5A* loss-of-function mutations. The cohort included families with *SCN5A* mutations leading to Brugada syndrome only and families with *SCN5A* mutations leading to either progressive cardiac conduction defects (PCCD) or Brugada syndrome [27]. These patients had a median QRS interval duration of 108±12 ms. This value was used as a cut-off to discriminate patients with minor conduction defects (QRS≤108 ms; mild phenotype) or major conduction defects (QRS>108 ms; severe phenotype). Non-mutation carrier siblings served as controls. Two age groups were then distinguished, 6–31 years and 41–61 years. In the 6–31 year-old patient group (mean value: 21±2 years; n = 19), patients with a mild phenotype had a mean QRS duration of 98±3 ms (80–108 ms; n = 12) and those with a severe phenotype had a mean QRS duration of 130±7 ms (110–158 ms; n = 7; p<0.001 *versus* mild). In the older patient group (51±1 years; n = 23), patients with a mild phenotype had a mean QRS interval of 94±2 ms (80–107 ms; n = 11) whereas patients with a severe phenotype had a QRS of 122±4 ms (112–160 ms; n = 12; p<0.001 *versus* mild). All patients were challenged with ajmaline according to current guidelines [28].

In a second study, clinical data from patients carrying *SCN5A* mutations leading to Brugada syndrome from the Nantes and Amsterdam databases were pooled (n = 108). This mixed database was used to discriminate symptomatic and asymptomatic patients. Symptomatic patients had a clinical history of collapses, syncopes, documented ventricular tachycardia (and/or fibrillation) or aborted sudden death. Baseline QRS interval duration were compared between symptomatic and asymptomatic patients.

### Histology

Cardiac arrest was induced in sedated mice (etomidate, 30 mg/kg i.p.) with intravenous infusion of 10% KCl. After excision, hearts were rinsed in saline solution and fixed by immersion in 10% formalin for 24h. Then, gross transverse sections were cut from the base to the apex and embedded in paraffin. Paraffin sections were stained with hematoxylin-eosin to assess any myocyte necrosis or interstitial inflammation or Sirius red to assess interstitial and scar fibrosis. Serial sections were cut to track the ventricular outflow tract. Semi-quantitative assessment of fibrosis was determined based upon the extent and the number of interstitial fibrosis and scar foci (and scored 0 to 3).

### TaqMan Real-Time RT-PCR

For *Atf3* and *Egr1* genes, Taqman real-time RT-PCR was performed as previously described [10]. The ion channel gene expression patterns of ventricular preparations from WT and *Scn5a* ^+/−^ mice were characterized with TaqMan Low Density Arrays (Applied Biosystems, Foster City, CA) in a two-step RT-PCR process. The genes selected for their cardiac expression encode 68 ion channel α- and auxiliary subunits, 7 proteins involved in calcium homeostasis, 5 transcription factors, specific markers of cardiac regions, vascular vessels, neuronal tissue, fibroblasts, inflammation and hypertrophy, and 4 endogenous control genes used for normalization. First strand cDNA was synthesized from 220 ng of total RNA using the High-Capacity cDNA Archive Kit (Applied Biosystems). Experimental procedure and data analysis were performed as previously described [29].

### Protein Isolation and Western Blot

After excision, hearts were rapidly frozen in liquid nitrogen and stored at −80°C. Frozen mouse ventricle was transferred into lysis buffer containing (in mmol/L): trishydroxyaminomethane (TRIS), 50 (pH 7.5); NaCl, 150; ethylenediaminetetra-acetic acid (EDTA), 1; phenylmethylsulfonyl fluoride (PMSF), 1; Complete® protease inhibitor cocktail from Roche Applied Science (Basel, Switzerland). Tissues were then homogenized using a Polytron. Triton Tx-100 was added to a final concentration of 1% and solubilization occurred by rotating for 1 h at 4°C. The soluble fraction from a subsequent 15-min centrifugation at 13,000 g (4°C) was used for the experiments. In order to load each lane of the SDS-page with equivalent amounts of total proteins, the protein concentration of each lysate was measured in triplicate by Bradford assay using a BSA standard curve.

Polyclonal anti-Na_v_1.5 antibody (ASC-005) was purchased from Alomone (Jerusalem, Israel) and rabbit anti-actin antibody (A 2066) was purchased from Sigma (Buchs, Switzerland). The immunoblot signals were visualized using enhanced chemiluminescence (ECL). Densitometric analyses of ECL films were performed using QuantityOne Software (BioRad, Hercules, California, USA). Bands were manually selected using the volume tool. For each band, the background was subtracted and the signal intensity was normalized to actin.

### Immunofluorescence Experiments on Isolated Cardiomyocytes

Cardiomyocytes were washed with phosphate-buffered saline (PBS, pH 7.4) and fixed in 2% paraformaldehyde (37°C). Cells were blocked/permeabilized in PBS containing 0.075% Triton X-100 and 3% fish oil gelatin, and incubated in primary antibody, (polyclonal Na_v_1.5 antibody) overnight at 4°C [30]. Following washes (PBS plus 0.1% Triton X-100), cells were incubated in secondary antibody (Alexa 568; Molecular Probes) for 8 hours at 4°C and mounted using Vectashield (Vector). Images were collected on a Zeiss 510 Meta confocal microscope (63 power oil immersion, 1.4 NA, 1.0 Airy Units) using Carl Zeiss Imaging software. Volocity Classification and LSM510 software (Zeiss) were used to measure pixel intensities. The following imaging protocol was adopted. Data were first collected on WT cardiomyocytes to establish a linear imaging protocol. All cardiomyocytes were imaged using an identical imaging protocol (LSM510 ‘re-use’ function to ensure consistency in pinhole diameter, brightness, contrast, and laser intensity). Since Na_v_1.5 is primarily expressed in the intercalated disc region [31], [32], we determined the mean staining intensity for 3 intercalated disc line scans per myocyte. For each group, at least 60 myocytes from 3 different mice were analyzed.

### Isolation of Cardiac Myocytes and Patch Clamp Experiments

Ventricular myocytes were isolated as previously described [33]. Whole-cell configuration of the patch-clamp technique was used to record I_Na_. Experiments were performed at room temperature (22–23°C), using a VE-2 (Alembic Instruments, Canada) amplifier allowing the recording of large Na^+^ currents. Pipettes (tip resistance of 1–2 MΩ) were filled with a solution containing (in mmol/L): CsCl, 60; Cesium asparte, 70; CaCl_2_, 1; MgCl_2_, 1; HEPES, 10; EGTA, 11; Na_2_ATP, 5 (pH was adjusted to 7.2 with CsOH). Myocytes were bathed with a solution containing (in mmol/L): NaCl, 10; NMDG-Cl, 120; CaCl_2_, 2; MgCl_2_, 1.2; CsCl, 5; HEPES, 10; Glucose 5 (pH was adjusted to 7.4 with CsOH). Holding potentials were −120 mV and I_Na_ densities (pA/pF) were obtained by dividing the peak I_Na_ by the cell capacitance. Peak currents were measured during a voltage-clamp protocol.

To quantify the voltage dependence of steady-state activation and inactivation, data from individual cells were fitted with the Boltzmann relationship, y (V_m_) = 1/1+exp[(V_m_−V)/K], in which y is the normalized current or conductance, V is the voltage at which half of the available channels are inactivated, K is the slope factor, and V_m_ is the membrane potential. The voltage dependence of inactivation was determined by measuring current in response to pulses (20 ms) to −20 mV that had been preceded by 500-ms pulses applied in a series of 5-mV incremental voltages from −130 mV to −20 mV.

### Microelectrode Studies

Seven wild-type (WT) and 11 *Scn5a* ^+/−^ (6 with a mild phenotype and 5 with a severe phenotype) adult mice of either sex were killed by cervical dislocation. The hearts were quickly removed and immersed in a cool modified Tyrode solution containing (in mmol/L): NaCl, 108; NaHCO_3_, 25; NaH_2_PO_4_, 1.8; KCl, 27; MgCl_2_, 1; CaCl_2_, 0.6; glucose, 55 and previously saturated with a 95% O_2_-5% CO_2_ gas mixture (pH 7.3). Preparations were mounted in a tissue bath chamber, the endocardial surface facing up and superfused with an oxygenated (95% O_2_-5% CO_2_) Tyrode solution warmed to 37±0.5°C and containing (in mmol/L): NaCl, 120; NaHCO_3_, 27; NaH_2_PO_4_, 1.2; KCl, 5.4; MgCl_2_, 1.2; CaCl_2_, 1.8; glucose, 10 (pH 7.4). Transmembrane recordings were obtained using standard methods. The tissues were allowed to recover for at least one hour before the experiments started. During this period, they were paced at a frequency of 300 beat per minute (bpm) by bipolar stimulation through Teflon-coated silver wire electrodes. Stimulus pulse width was 1 ms and amplitude was twice diastolic threshold. The preparations were then paced at frequencies increasing from 200 to 800 bpm. Action potentials characteristics were measured at steady state for each frequency.

### Statistical Analysis

Data are expressed as mean ± SEM. Statistical analysis was performed with Student *t*-test, Kruskall-Wallis test and one- or two-way analysis of variance completed by a Tukey test when appropriate. The Fisher Exact test was used for the statistical analysis of proportions of mice with arrhythmias. A value of p<0.05 was considered significant.
